# miR-541 serves as a prognostic biomarker of osteosarcoma and its regulatory effect on tumor cell proliferation, migration and invasion by targeting TGIF2

**DOI:** 10.1186/s13000-020-01008-9

**Published:** 2020-07-24

**Authors:** Chunlei Liu, Xiuling Yi

**Affiliations:** Department of Spinal Surgery, Weifang People’s Hospital, No. 151 Guangwen Street, Weifang, 261000 Shandong China

**Keywords:** Osteosarcoma, microRNA-541, Prognosis, Proliferation, Migration, Invasion, TGF-β-induced factor homeobox 2

## Abstract

**Background:**

Several studies reported the dysregulation of miR-541 in the progression of some human malignancies. Osteosarcoma (OS) is one of the most common primary malignant bone tumors. This study aimed to assess the expression and clinical significance of miR-541 in OS patients and explore the biological function of miR-541 in tumor progression.

**Methods:**

Expression of miR-541 was detected by quantitative real-time PCR, and its prognostic value was evaluated using Kaplan-Meier survival analysis. The biological function of miR-541 was examined by analyzing its effects on OS cell proliferation, migration and invasion. Additionally, the underlying potential target of miR-541 was predicated and analyzed.

**Results:**

The expression of miR-541 was significantly decreased in OS tissues and cell lines. The deregulated expression of miR-541 in tumor tissues was associated with the overall survival of OS patients and was a potential independent prognostic indicator. In OS cells, the overexpression of miR-541 could inhibit cell proliferation, migration and invasion. The luciferase activity results indicated that TGIF2 was a potential target of miR-541.

**Conclusion:**

The results of this study revealed that the decreased miR-541 expression in OS patients may serve as a prognostic biomarker, and that the overexpression of miR-541 in OS cells results in inhibited cell proliferation, migration and invasion, indicating the potential of miR-541 as a therapeutic target in OS treatment.

## Introduction

Osteosarcoma (OS) is one of the most common primary malignant bone tumors, which represents the leading cause of cancer death [1]. It is predominant in the distal femur, the proximal, and the proximal humerus, it also occurs in any human bone [2]. The mechanism of the formation and progression has been paid specific attention, the 5-year survival rate was only 30% [3]. Recently, significant progress has been made in the therapeutic strategies for the treatment of osteosarcoma, but the prognosis of patients with osteosarcoma is still poor [4]. Therefore, it is essential to develop a novel biomarker, which can provide therapeutic strategies and improve the prognosis of OS.

MicroRNAs (miRNA) are a series of non-coding RNAs, containing 18–21 nucleotides, which are involved in the regulation of gene expression at the post-transcriptional level [5]. Overwhelming evidence reported that miRNAs play important roles in some biological processes, such as cell proliferation, migration, invasion, differentiation and apoptosis [6]. In addition, numerous studies have found a number of miRNAs with aberrant expression in cancer tissues and cells, indicating their association with the development of cancers [7, 8]. The deregulated expression of miRNAs attracts increasing attention on their significantly clinical significance in cancer diagnosis and prognosis [9]. Thus, identifying novel miRNAs that play functional role in tumor progression may help to improve the treatment of OS.

MicroRNA-541 (miR-541) has been reported to be dysregulated in a number of cancers and tumors. In non-small cell lung cancer, miR-541 expression was found to be decreased in tumor tissues and cells, and its inhibiting effect on tumor cell proliferation and migration was further demonstrated [10]. In hepatocellular carcinoma, the downregulated expression of miR-541 was also been found in tumor tissues and cells, and the tumor cell invasion and migration could be suppressed by miR-541 [11]. In OS, Patricia et al. has investigated the deregulated miRNAs in pediatric OS by in silico analysis, and predicted that miR-541 might be associated with OS overall survival [12]. However, the role of miR-541 in OS remains unclear due to the lack of experimental verification.

In this study, we sought to assess the expression of miR-541 in OS tissues and cells, evaluate the prognostic value of miR-541 in OS patients and explore the biological function of miR-541 in OS progression. The results of this study may provide a novel biomarker for OS prognosis and a potential therapeutic target for OS treatment.

## Material and methods

### Patients and tissue samples collection

The research was performed with the approval of the Ethics Committee of Weifang People’s Hospital. OS tissues and adjacent normal tissues were collected from OS 106 patients between January 2009 to December 2013 at Weifang People’s Hospital. All the collected tissues have been diagnosed by at least two pathologists and the clinical stages were determined using the criteria by the Musculoskeletal Tumor Society (MSTS) system [13]. All of the tumor tissues were determined as high grade, and no low grade cases were included due to the low incidence rate. The written informed consent was obtained from each patient before sampling. No patients had received any anti-tumor therapy, and the clinicopathological features of the patients are summarized in Table 1. The collected tissues were frozen in liquid nitrogen until further analysis. In addition, a 5-year follow-up survey was carried out to get the survival information of the OS patients.

**Table 1:** Association between miR-541 expression and clinical features in OS patients

Parameters	Cases No. (*n* = 106)	miR-541-low (*n* = 62)	miR-541-high (*n* = 44)	*P* value
Gender				
Male	64	38	26	0.861
Female	42	24	18	
Age				
< 18	60	32	28	0.970
≥ 18	46	30	16	
Anatomical location				
Tibia/femur	66	37	29	0.808
Fibula	24	15	9	
Humerus	16	10	6	
Tumor size (cm)				
< 8	55	26	29	0.015
≥ 8	51	36	15	
Enneking stage				
I-IIB	41	20	21	0.005
IIB-III	65	42	23	
Distant metatasis				
Negative	50	22	28	0.004
Positive	56	40	16	
Histological subtype				
Osteoblastic	62	35	27	0.501
Chondroblastic	24	13	11	
Fibroblastic	20	14	6	
Histological gradeTumor differentiation				
Well/moderate	61	29	32	0.008
Poor	45	33	12	

### Cell lines and transfection

Four OS cell lines MG63, U2OS, HOS, and SAOS-2 and a normal cell line hFOB 1.19 were purchased from the Chinese Academy of Sciences Cell Bank (Shanghai, China). The cells were cultured in RPMI1640 medium (Gibco, El Paso, TX, USA) with 10% fetal bovine serum (FBS) in a humidified incubator at 37 °C with 5% CO_2_. To regulate the expression of miR-541 in U2OS and HOS cells, the cells were transfected with miR-541 mimic or mimic negative control (NC) (RiboBio, Guangzhou, China by Lipofectamine 2000 reagent (Invitrogen, Carlsbad, 125 CA, USA) according to the manufacturer’s instruction.

### RNA extraction and quantitative real-time PCR (qRT-PCR)

Total RNA including miRNA was extracted from tissue samples and cells with the help of TRIzol reagent (Invitrogen, Carlsbad, CA, USA), and was purified with a miRNeasy mini kit (Qiagen, Hilden, 133 Germany). The single stranded cDNA was synthesized from RNA using a PrimeScript™ RT reagent kit (Takara, Dalian, China). qPCR was performed using the SYBR Green I Master Mix kit (Invitrogen, Carlsbad, CA, USA) and the Applied Biosystems 7500 Fluorescent Quantitative PCR system (Applied Biosystems Life Technologies, USA). U6 was used to normalize the expression levels of miR-541. The 2^-ΔΔCt^ method was used to determine relative quantitation of miR-541.

### Cell proliferation assay

The cell counting kit-8 (CCK-8) assay and colony formation assay were employed to evaluate the cell proliferation of the transfected cells. After 48 h of cell transfection, the cells were seeded into 96-well plates with the concentration of 5000 cells per well. At 0 h, 24 h, 48 h and 72 h, 10 μL CCK-8 reagent (Dojindo, Kumamoto, Japan) was added to each well. After that, the cells were incubated at 37 °C with 5% CO_2_ for 4 h. The absorbance at 450 nm was measured to evaluate the proliferation of OS cells.

For the colony formation assay, the cells were plated in 6-well plates with the density of 500 cells/well and incubated at 37 °C for 14 days. After the incubation, cells in each well were washed with PBS and fixed with methanol and stained with Giemsa for 10 min. The number of cell colonies were calculated to estimate the proliferation ability of OS cells.

### Cell migration and invasion assay

The migration and invasion abilities of OS cells were evaluated using Transwell chambers (Corning) with a pore size of 8 μm. The chambers used for invasion assay were pre-coated with Matrigel, but the chambers for migration assay had no need for precoating. The transfected cells with a density of 1 × 10^4^ cells/well were seeded into the upper chamber in serum-free medium, and the lower chambers contained medium supplemented with 10% FBS as a chemoattractant. At 24 h following the incubation, cells in the lower chamber were fixed in 70% ethanol for 30 min and stained with 0.2% crystal violet for 10 min at room temperature. The cell numbers in 5 random visual fields were counted by an inverted microscope (Olympus, Tokyo, Japan).

### Luciferase reporter assay

The potential targets of miR-541 were predicted by TargetScan, and TGF-β-induced factor homeobox 2 (TGIF2) was found to contain a complementary sequence of miR-541. The luciferase reporter assay was subsequently performed to confirm the interaction between miR-541 and TGIF2. U2OS and HOS cells were seeded in 6-well plates and cultured for 24 h. Then the cells were co-transfected with miR-NC or miR-541 and luciferase reporter containing the wild type (WT) or mutant type (MT) of TGIF2 3′-UTR by Lipofectamine 2000 reagent (Invitrogen, Carlsbad, CA, USA) according to the manufacturer’s instruction. After 24 h post-transfection, the Dual-Luciferase Reporter Assay System (Promega, Madison, Wisconsin, USA) was used to analyze the luciferase activity.

### Statistical analysis

All data were present as mean ± standard deviation (SD) and analyzed by SPSS 21.0 software (SPSS, Inc., Chicago, IL, USA) and GraphPad Prism 7.0 software (GraphPad Software, Inc., Chicago, USA). Comparisons between groups were assessed by Student’s t test, Chi-square test or one-way ANOVA followed by Tukey’s test. Kaplan-Meier analysis and Cox regression analysis were employed to analyze the prognostic significance of miR-541-3p. The differences were considered to be significant when *P* < 0.05.

## Results

### Downregulated expression of miR-541 in OS tissues and cell lines

**Fig. 1:**
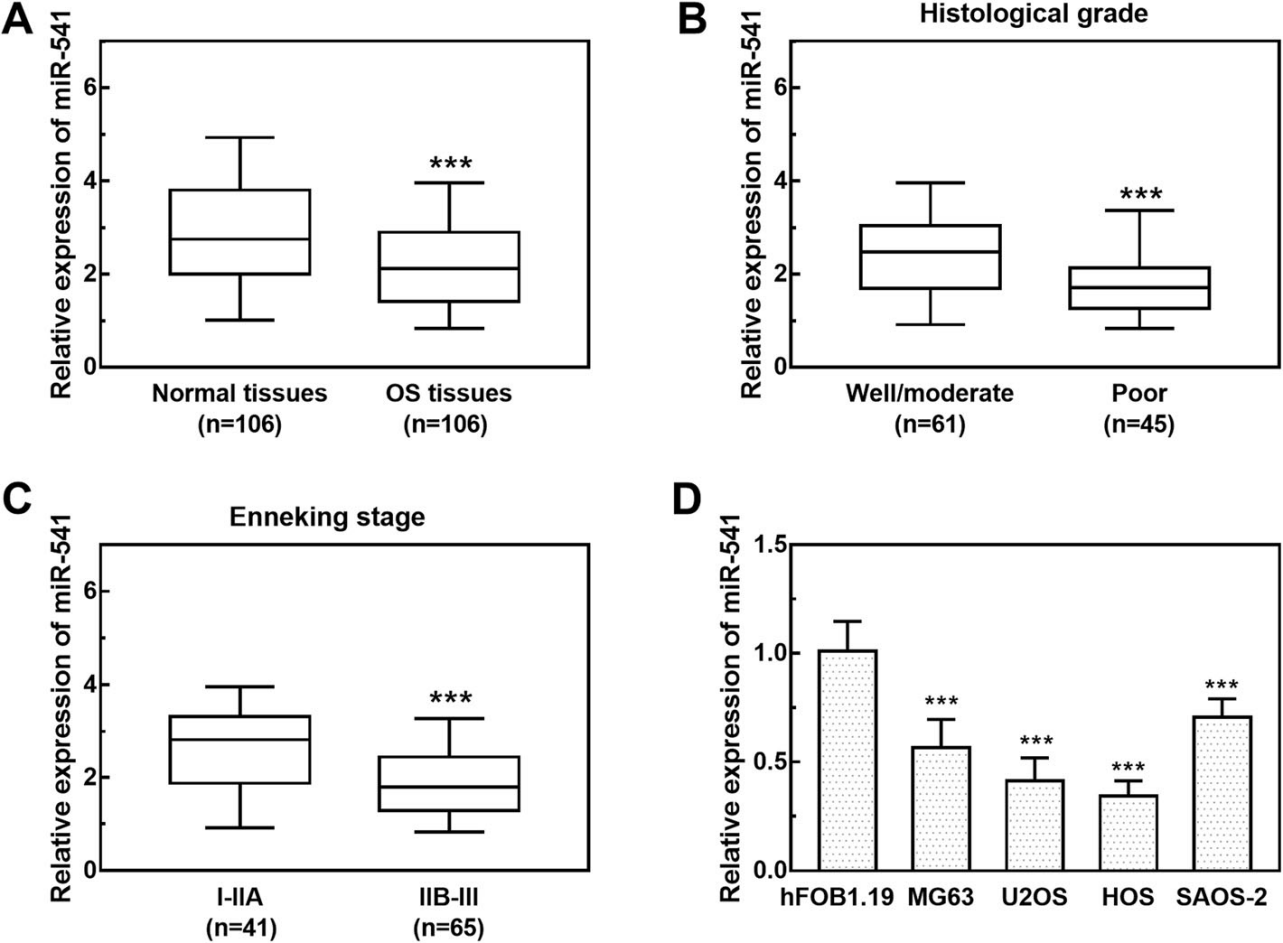
miR-541 was downregulated in human OS tissues and cell lines. **a** The expression of miR-541 was significantly decreased in OS tissues compared with adjacent normal tissues (***P < 0.001). **b** The expression of miR-541 was reduced in patients with poor differentiation (***P < 0.001). **c** The expression of miR-541 was downregulated in patients with advanced Enneking stage compared with those with early stage (***P < 0.001). (D) The expression of miR-541 was significantly decreased in OS cell lines compared with normal cells (***P < 0.001)

The expression of miR-541 was analyzed with the help of qPCR, and the results were summarized in Fig. 1. miR-541 was significantly downregulated in OS tissues, compared with adjacent normal tissues (*P* < 0.001, Fig. 1a). In addition, the expression of miR-541 was lower in patients with poor differentiation tumors than those with well/moderate differentiation tumors (*P* < 0.001, Fig. 1b). By staging according to the MSTS system, 41 patients were determined as Enneking I-IIA stage and 65 as IIB-III stage. The patients with advanced clinical stage had significantly decreased miR-541 expression compared with the patients with early stage (*P* < 0.001, Fig. 1c). Consistently, in four OS cell lines (MG63, U2OS, HOS, SAOS-2), the expression of miR-541 was markedly lower than that in the normal cell hFOB1.19 (all *P* < 0.001, Fig. 1d). Additionally, the expression of miR-541 in U2OS and HOS cells was relatively lower than the other two cells, therefore, U2OS and HOS were chosen for the following cell experiments.

### Association between expression of miR-541 and the clinicopathological characteristics of OS patients

The 106 patients contained 64 males and 42 females with an average age of 19.28 ± 8.23 years, and 56 cases had lung metastasis at diagnosis. To facilitate the relationship analysis between miR-541 and the clinical feature, OS patients were divided into two groups based on the median expression of miR-541, including high expression group (*n* = 62) and low expression group (*n* = 44), and the association between miR-541 expression and the clinicopathological features was evaluated. As shown in Table 1, the expression of miR-541 was closely related to the tumor size (*P* = 0.015), Enneking stage (*P* = 0.005), distant metastasis (*P* = 0.004) and tumor differentiation (*P* = 0.008). While other characteristics of patients, including age, gender, anatomical location and histological subtypes, showed an insignificant association with the expression of miR-541 (*P* > 0.05).

### Prognostic value of miR-541 in patients with OS

**Fig. 2:**
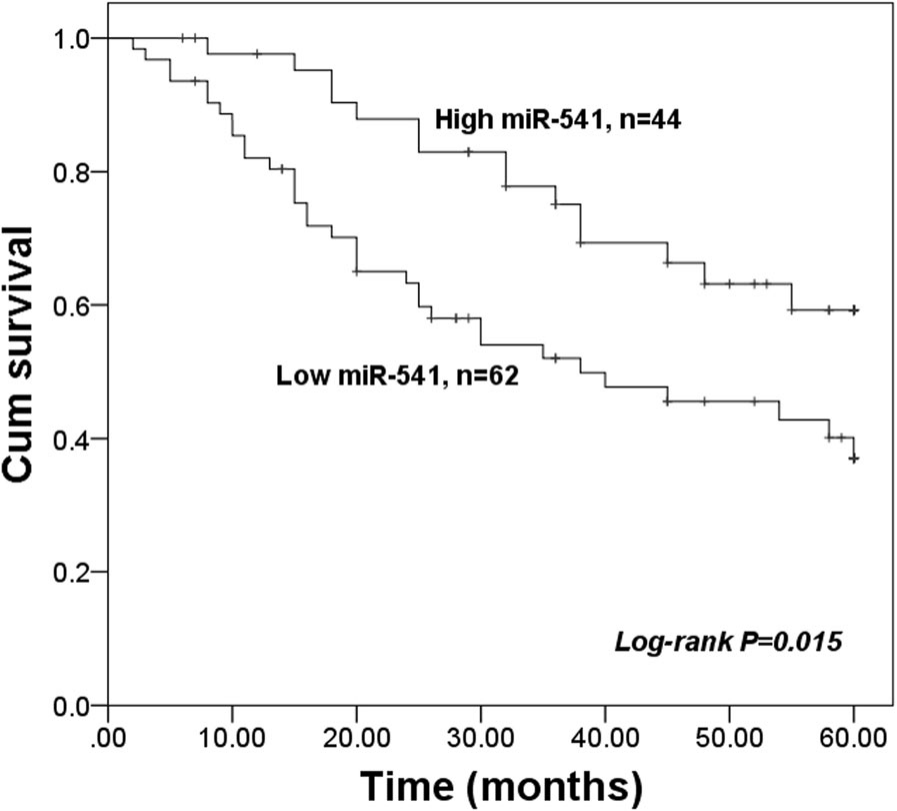
Survival curves for OS patients constructed by the Kaplan-Meier method. Patients with low miR-541 expression had a shorter survival time compared with those with high miR-541 expression. Log-rank *P* = 0.015

The 5-year follow-up survival information was used to construct the survival curves of OS patients using Kaplan-Meier method. As shown in Fig. 2, the patients with low miR-541 expression had shorter survival time compared with those with high miR-541 expression (log-rank *P* = 0.015). In addition, the Cox regression analysis data listed in Table 2 revealed that the expression of miR-541 was an independent prognostic factor (*P* = 0.006) with the HR of 2.559 (95% CI = 1.312–5.012).

**Table 2:** Cox regression analysis in the patients with OS

Variables	Univariate analysis			Multivariate analysis		
	HR	95% CI	*P* value	HR	95% CI	*P* value
miR-541 low vs. high	2.612	1.523–5.845	0.004	2.599	1.312–5.012	0.006
Gender male vs. female	1.139	0.637–2.038	0.661	1.521	0.812–2.882	0.265
Age (years) ≥ 18 vs. < 18	1.124	0.640–1.975	0.684	1.234	0.575–2.127	0.713
Anatomical location tibia/femur vs. others	1.289	0.644–1.812	0.495	1.185	0.601–1.985	0.812
Tumor size (cm) ≥ 8 vs. < 8	1.520	0.867–2.665	0.144	1.321	0.685–2.254	0.451
Enneking stage IIB-III vs. I-IIA	2.412	1.428–5.625	0.008	2.212	1.198–4.114	0.016
Distant metastasis positive vs. negative	2.375	1.321–5.286	0.010	2.352	1.210–4.285	0.012
Histological subtype osteoblastic/chondroblastic vs. fibroblastic	1.485	0.856–2.458	0.128	1.521	0.721–2.386	0.324
Histological gradeTumor differentiation poor vs. well/moderate	1.968	1.089–4.996	0.039	1.985	1.085–3.891	0.046

### Effect of miR-541 on cell proliferation, migration and invasion of OS cells

**Fig. 3:**
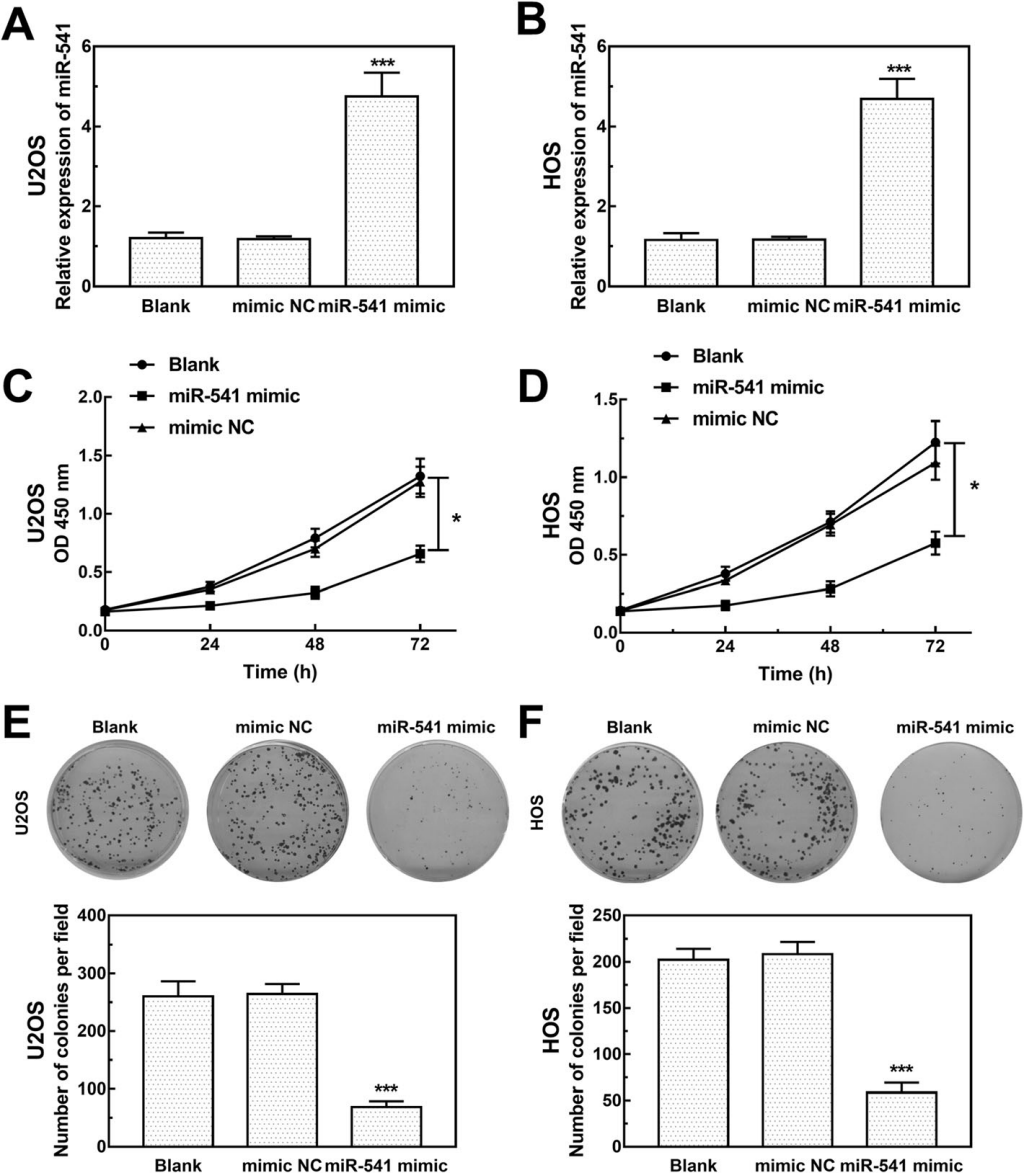
Effects of miR-541 on OS cell proliferation, migration, and invasion of OS. **a** and **b** The expression of miR-541 in U2OS and HOS transfected with miR-541 mimic or miR-541 mimic NC (***P < 0.001). **c** and **d** Cell proliferation of U2OS and HOS transfected with miR-541 mimic or miR-541 mimic NC (*P < 0.05). **e** and **f** Colony formation assay results for U2OS and HOS cells transfected with miR-541 mimic or mimic NC (***P < 0.001)

**Fig. 4:**
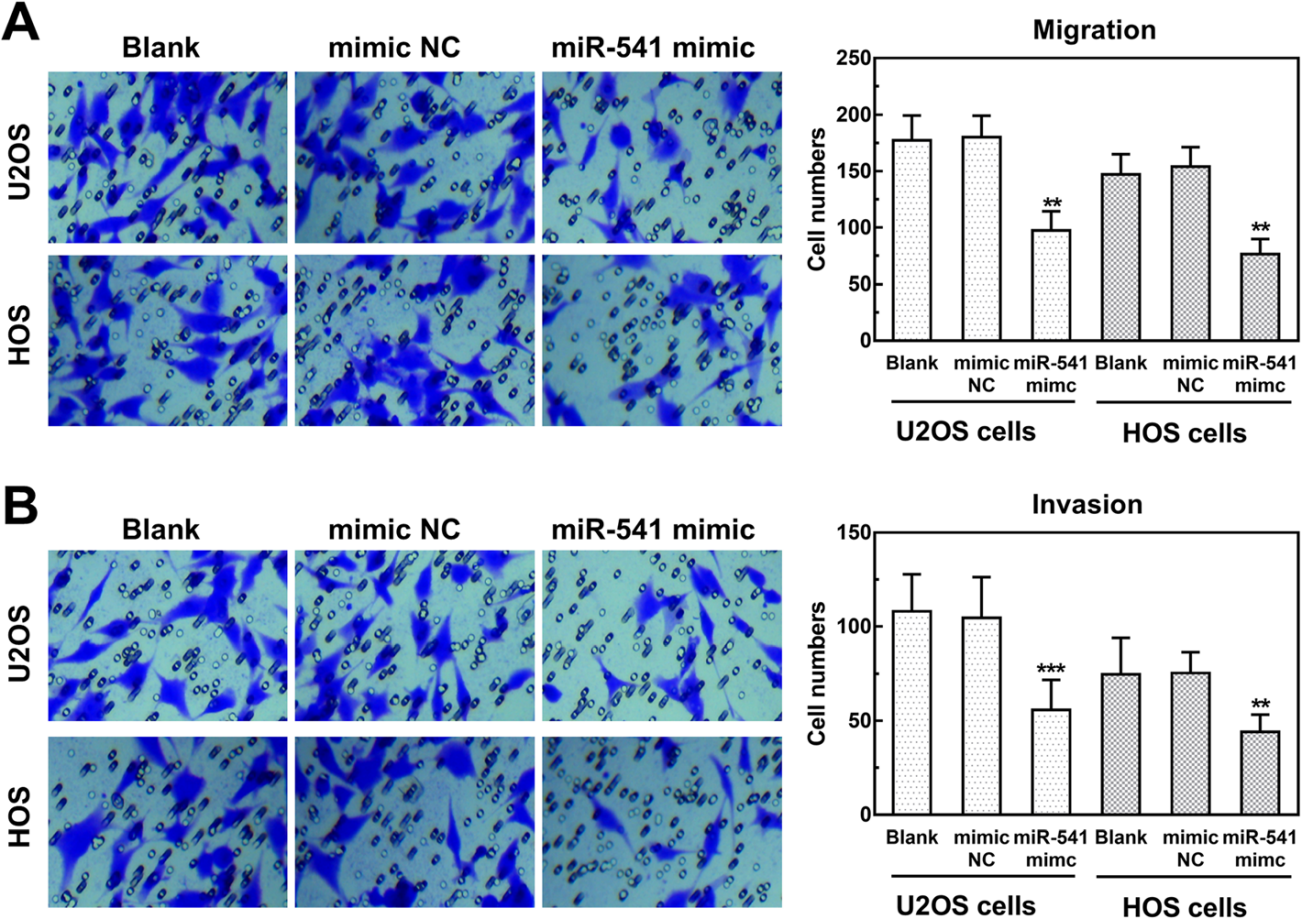
Effect of miR-541 on OS cell migration and invasion. **a** Cell migration of U2OS and HOS transfected with miR-541 mimic or mimic NC (**P < 0.01). **b** Cell invasion of U2OS and HOS transfected with miR-541 mimic or mimic NC (**P < 0.01, ***P < 0.001)

U2OS and HOS cells were transfected with miR-541 mimic or mimic NC to regulate the expression of miR-541 in the cells. As shown in Fig. 3a and b, the expression of miR-541 was significantly upregulated in both two cell lines transfected with miR-541 mimic (both *P* < 0.001). The results of CCK8 assay and cell colony formation assay showed that the cell proliferation was significantly inhibited by the overexpression of miR-541 in both U2OS and HOS cells (*P* < 0.05, Fig. 3c – f). Moreover, the migration and invasion of the two cell lines were measured by Transwell assay. The results indicated that the overexpression of miR-541 significantly inhibited OS cell migration and invasion (all *P* < 0.01, Fig. 4).

### TGIF2 is a direct target of miR-541

**Fig. 5:**
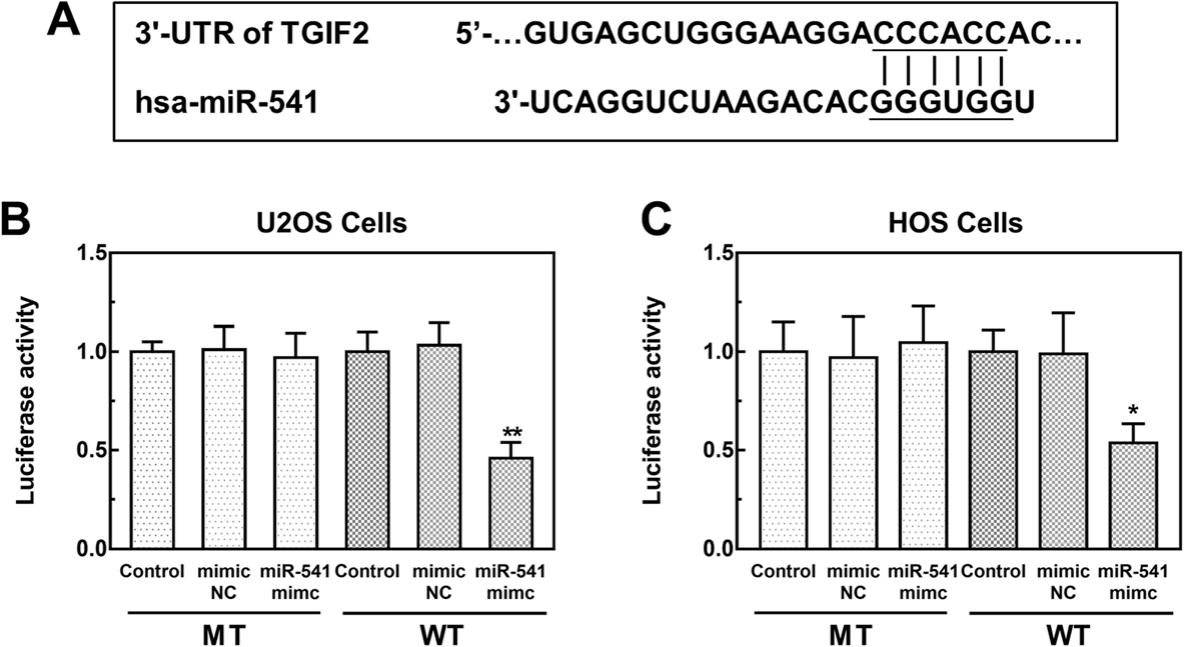
TGIF2 is a potential target of miR-541. **a** Complementary sequences of miR-541 at the 3′-UTR of *TGIF2*. **b** and **c** Luciferase activity in U2OS and HOS cells (*P < 0.05, **P < 0.01)

A complementary sequence of miR-541 was found in the 3′-UTR of TGIF2 (Fig. 5a). The following luciferase reporter assay results shown in Fig. 5b and c revealed that the relative luciferase activity in WT group was markedly decreased by the upregulation of miR-541 in both the U2OS and HOS cell lines (both *P* < 0.05), while no significant changes were observed in the luciferase activity in MT group (*P* > 0.05), indicating the interaction between miR-541 and TGIF2 in OS cells.

## Discussion

OS is a kind of common primary malignant bone tumor, which possesses a poor survival rate and high recurrence and metastasis rates [14]. Although the prognosis of OS has been improved with the development of therapeutic strategies, there is also an urgent need for more novel biomarkers for the treatment of OS [15]. Recently, the role of miRNAs in the progression of various human malignancies has attracted huge attention [16, 17]. More and more miRNAs have been reported to be associated with the occurrence of tumor, especially the miRNAs with abnormal expression [18, 19]. For example, miR-125 was upregulated in colorectal cancer, and it exerted inhibitory effects on the proliferation and invasion of colorectal cancer cells [20]. In non-small cell lung cancer, miR-1269a was upregulated and it acted as an onco-miRNA in NSCLC and promotes cancer cell growth through downregulating the expression of SOX6 [21]. Additionally, there are also various miRNAs reported to play vital roles in the development and progression of OS, such as miR-98-5p, miR-197, miR130b-5p [22–24]. Therefore, the miRNAs with dysregulation in OS have drawn special attention.

Several studies have demonstrated the functional role of miR-541 in some cancers [25]. For instance, miR-541 targeted the cell cycle regulator CCND1, which resulted in the inhibition of cell proliferation and progression of prostate cancer [26]. miR-541 also has been demonstrated to play a role in the development of breast cancer and lung cancer [25, 27]. In this research, we found that miR-541 was significantly downregulated in OS tissues and cell lines, and the expression of miR-541 was closely associated with tumor size, clinical stage, distant metastasis and tumor differentiation of OS patients. These results indicated that the aberrant expression of miR-541 might be involved in the development of OS. In addition, this study evaluated the clinical significance of miR-541 in the prognosis of OS. Some miRNAs have been proposed as potential prognostic biomarkers in OS. For example, serum decreased expression of miR-124 has been reported to be candidate diagnostic and prognostic biomarker of OS [28]. The downregulation of miR-1225-5p in OS was associated with poor prognosis. Of note, the decreased miR-541 has been determined to be a prognostic biomarker in patients with non-small cell lung cancer [10]. In the present study, the survival curves showed that OS patients with low miR-541 had a poor prognosis, and the Cox regression data demonstrated that miR-541 might be an independent prognostic indicator for the overall survival of OS patients.

Accumulated evidence has demonstrated the important regulatory effect of miRNAs on cell processes in various tumor cells [17, 29]. miR-541 has been reported to inhibit tumor progression by suppressing tumor cell proliferation, migration and invasion in prostate cancer [26] and squamous cell lung carcinoma [25]. In this study, the expression of miR-541 in OS cells was upregulated by cell transfection, and we found that the OS cell proliferation, migration and invasion were significantly inhibited by the overexpression of miR-541. Thus, we considered that miR-541 might serve as a tumor suppressor in OS progression.

TGIF2 has been identified as an oncogene in some cancers, such as lung adenocarcinoma [30] and glioma [31]. In OS, TGIF2 has also been reported to be involved in tumor progression [32]. This study predicted that TGIF2 was a potential target of miR-541 by bioinformatics analysis, and further confirmed their interaction in OS cells. In a previous study by Lu et al. reported that miR-541 inhibited non-small cell lung cancer cell proliferation and migration by targeting TGIF2 [10]. The conclusion of this study combined with our analysis results led us to deduce that miR-541 might also be involved in the progression of OS by targeting TGIF2. Although this study provides some evidence for the role of miR-541 in OS, the precise mechanisms underlying the effect of miR-541 on OS progression need to be confirmed and deeply analyzed in further investigations.

## Conclusion

Taken together, this study demonstrated that miR-541 expression is downregulated in OS tissues and cell lines. The expression of miR-541 is significantly associated with tumor size, clinical stage, distant metastasis and tumor differentiation of OS patients, and serves as a candidate prognostic biomarker. The overexpression of miR-541 can inhibit OS cell proliferation, migration and invasion, indicating the tumor suppressor role of miR-541 in OS progression. This study provides a novel biomarker and a potential therapeutic target in the treatment of OS. The methods to increase miR-541 expression may help to improve the therapeutic strategies of OS.

## Data Availability

All data generated or analyzed during this study are included in this published article.

## Abbreviations

OS: Osteosarcoma
miRNAs: microRNAs
miR-541: microRNA-541
TGIF2: TGF-β-induced factor homeobox 2
CCK-8: Cell Counting Kit-8
SD: Standard deviation
